# Anti-Inflammatory Effects of Pomegranate Peel Extract in THP-1 Cells Exposed to Particulate Matter PM10

**DOI:** 10.1155/2016/6836080

**Published:** 2016-05-10

**Authors:** Soojin Park, Jin Kyung Seok, Jun Yup Kwak, Hwa-Jin Suh, Young Mi Kim, Yong Chool Boo

**Affiliations:** ^1^Department of Molecular Medicine, Cell and Matrix Research Institute, BK21 Plus KNU Biomedical Convergence Program, School of Medicine, Kyungpook National University, 680 Gukchaebosang-ro, Jung-gu, Daegu 41944, Republic of Korea; ^2^Gyeongbuk Natural Color Industry Institute, 181 Cheonmun-ro, Yeongcheon-si, Gyeongsangbuk-do 38896, Republic of Korea; ^3^Ruby Crown Co., Ltd., 201 Kyungpook National University Business Incubation Center, 80 Daehak-ro, Buk-gu, Daegu 41566, Republic of Korea

## Abstract

Epidemiological and experimental evidence support health risks associated with the exposure to airborne particulate matter with a diameter of <10 *μ*M (PM10). PM10 stimulates the production of reactive oxygen species (ROS) and inflammatory mediators. Thus, we assumed that natural antioxidants might provide health benefits attenuating hazardous effects of PM10. In the present study, we examined the effects of pomegranate peel extract (PPE) on THP-1 monocytic cells exposed to PM10. PM10 induced cytotoxicity and the production of ROS. It also increased the expression and secretion of inflammatory cytokines, such as tumor necrosis factor-*α* (TNF-*α*), interleukin-1*β* (IL-1*β*), and monocyte chemoattractant protein-1 (MCP-1), and cell adhesion molecules, such as intercellular adhesion molecule-1 (ICAM-1) and vascular cell adhesion molecule-1 (VCAM-1). PPE at 10–100 *μ*g mL^−1^ attenuated the production of ROS and the expression of TNF-*α*, IL-1*β*, MCP-1, and ICAM-1, but not VCAM-1, in THP-1 cells stimulated by PM10 (100 *μ*g mL^−1^). PPE also attenuated the adhesion of PM10-stimulated THP-1 cells to EA.hy926 endothelial cells. PPE constituents, punicalagin and ellagic acid, attenuated PM10-induced monocyte adhesion to endothelial cells, and punicalagin was less cytotoxic compared to ellagic acid. The present study suggests that PPE and punicalagin may be useful in alleviating inflammatory reactions due to particulate matter.

## 1. Introduction

Air pollution has become the world's largest single environmental health risk [1]. Major outdoor air pollutants include particulate matter, volatile organic compounds, and hazardous gases. Airborne particulate matter is a fine dust of natural and artificial origins suspended in the Earth's atmosphere. Natural particulates originate from volcanoes, dust storms, forest and land fires, and so on. Significant amounts of particulates are also generated from human activities, such as the burning of fossil fuels in vehicles, power plants, and various industrial processes. Many previous studies have demonstrated that a large number of deaths and other health problems were associated with particulate pollution [1–3].

Particulate matter is known to cause airway epithelium injury and endothelial dysfunction [4, 5]. Larger particles can be filtered in the nose and throat via cilia and mucus, but particulate matter smaller than 10 micrometers (PM10) can enter the deepest parts of the lungs, such as the bronchioles and alveoli [6]. PM10 may cause severe effects on human health due to the broad range of miscellaneous toxic compounds present in this particulate matter fraction, such as transition metals, endotoxins, and ultrafine components. The mechanism of action of PM10 may include the induction of oxidative stress and activation of nuclear factor kappa B (NF-*κ*B) pathway, leading to inflammation [7]. PM10 increases the production of reactive oxygen species (ROS) and cytokines in human and rat alveolar macrophages [8]. In addition, PM10-induced inflammation is attenuated by antioxidants from plant sources [9–11].

The pomegranate (*Punica granatum* L.) is a deciduous fruit tree belonging to the family Lythraceae. The rind of the fruit and the bark of the pomegranate tree have been used in traditional medicine for the treatment of diarrhea, dysentery, and intestinal parasites. Today, pomegranate juice is a popular drink worldwide. The major phytochemicals in pomegranate are ellagitannins, including punicalagin [12], which is a good antioxidant with potent free-radical scavenging properties [13]. Previous studies have examined the effects of pomegranate juice on various cardiovascular risk factors, including low density lipoprotein oxidation, macrophage oxidative status, foam cell formation, and high blood pressure [14–16]. However, no previous studies have examined the effects of pomegranate extracts on the cellular response to particulate matters.

In the present study, we hypothesized that pomegranate peel extract (PPE) may attenuate oxidative stress and inflammatory events induced by PM10. Therefore, we monitored the production of ROS and the expression of inflammatory cytokines and cell adhesion molecules in THP-1 monocytic cells exposed to PM10 in the absence and presence of PPE. Effects of PPE on the cell-cell adhesion between PM10-stimulated THP-1 cells and EA.hy926 endothelial cells were also examined.

## 2. Materials and Methods 

### 2.1. Reagents

Punicalagin (purity > 98%, a mixture of 40%  *α* and 60%  *β* anomers) and ellagic acid (purity > 98%) were purchased from Sigma-Aldrich (St. Louis, MO, USA). Fine dust (PM10-like) (European reference material ERM-CZ120) was purchased from Sigma-Aldrich. PPE was obtained from Hwasoomok Co. (Youngchen, Korea). The extract was prepared by extracting dry raw materials with water at 55°C for 2 h, followed by concentration and spray drying.

### 2.2. High Performance Liquid Chromatography (HPLC) Analysis

HPLC analysis was performed using a Gilson HPLC system (Gilson, Inc., Middleton, WI, USA) equipped with an ultraviolet/visible (UV/VIS) 151 detector. The volume of sample injected was 20 *μ*L, and separation was performed on a 5 *μ*m Hector-M C18 column (4.6 mm × 250 mm) (RS Tech Co. Daejeon, Korea) using a mobile phase consisting of 1% formic acid in water (A) and 1% formic acid in acetonitrile (B). A linear gradient from 0% to 30% B for 40 min and 30% to 100% 40–45 min was applied. The flow rate of the mobile phase was 0.6 mL min^−1^. The detector was set at 254 nm.

### 2.3. Cultivation of THP-1 Cells

THP-1 cells (human acute monocytic leukemia cell line) were obtained from the Korea Cell Line Bank (Seoul, Korea) and cultured in T-75 flasks (Nunc, Roskilde, Denmark) in an upright position. Culture medium was Roswell Park Memorial Institute (RPMI) 1640 medium (Gibco BRL, Grand Island, NY, USA) containing fetal bovine serum (10%), antibiotics (100 U mL^−1^ penicillin, 100 *μ*g mL^−1^ streptomycin, and 0.25 *μ*g mL^−1^ amphotericin B), and *β*-mercaptoethanol (0.05 mM). Cell viability was assessed using the trypan blue exclusion assay.

### 2.4. PM10 Treatments

THP-1 cells were seeded on 12-well plates at 4 × 10^5^ cells cm^−2^ and treated with PM10 at 3–100 *μ*g mL^−1^ for 24 h. In some experiments, cells were treated with PM10 (100 *μ*g mL^−1^) in the presence of test materials for the indicated time.

### 2.5. Assay for ROS Production

Cellular production of ROS was determined using dihydrorhodamine 123 (DHR123) (Sigma-Aldrich). THP-1 cells were treated with PM10 in the absence or presence of PPE for 24 h. Cells were labeled with 1.0 *μ*M DHR123 for the last 6 h of PM10 treatment. The oxidized rhodamine 123 was extracted from cells using ice-cold 70% ethanol/0.1 N HCl, followed by centrifugation at 13,000 rpm for 15 min. The supernatants were neutralized with 1 M NaHCO_3_ and spun down to obtain clear supernatants. Fluorescence intensity of the supernatants was measured at an excitation wavelength at 485 nm and an emission wavelength of 590 nm, using the Gemini EM fluorescence microplate reader (Molecular Devices, Sunnyvale, CA, USA).

### 2.6. Quantitative Reverse-Transcriptase Polymerase Chain Reaction (qRT-PCR) Analysis of Cytokine Expression 

THP-1 cells were treated with PM10 in the absence or presence of PPE for 24 h. Cellular RNA was extracted from the treated cells with an RNeasy kit (Qiagen, Valencia, CA, USA). One microgram of cellular mRNA was reverse transcribed to prepare complementary DNA (cDNA), using a High Capacity cDNA Archive Kit (Applied Biosystems, Foster City, CA, USA). PCR was conducted with a StepOnePlus*™* Real-Time PCR System (Applied Biosystems) in a reaction mixture (20 *μ*L) containing SYBR® Green PCR Master Mix (Applied Biosystems), 60 ng of cDNA, and 2 pmol of gene-specific primer sets (Macrogen, Seoul, Korea). The primers used were as follows: tumor necrosis factor-*α* (TNF-*α*) (GenBank accession number, NM_000594.3) 5′-TGC TCC TCA CCC ACA CCA T-3′ (forward) and 5′-GAG ATA GTC GGG CCG ATT GA-3′ (reverse); interleukin-1*β* (IL-1*β*) (NM_000576.2) 5′-CCT GTC CTG CGT GTT GAA AGA-3′ (forward) and 5′-GGG AAC TGG GCA GAC TCA AA-3′ (reverse); monocyte chemoattractant protein-1 (MCP-1) (MCP-1) (NM_002982.3) 5′-GCA ATC AAT GCC CCA GTC A-3′ (forward) and 5′-TGC TTG TCC AGG TGG TCC AT3′ (reverse); intercellular adhesion molecule 1 (ICAM-1) (NM_000201.2) 5′-ATC TGT GTC CCC CTC AAA AGT C-3′ (forward) and 5′-TGG CTA TCT TCT TGC ACA TTG C-3′ (reverse); vascular cell adhesion molecule 1 (VCAM-1) (NM_001079.3) 5′-CTG ACC CTG AGC CCT GTG A-3′ (forward) and 5′-CTT ACA GTG ACA GAG CTC CCA TTC-3′ (reverse); glyceraldehyde-3-phosphate dehydrogenase (GAPDH) (NM_002046.3) 5′-ATG GGG AAG GTG AAG GTC G-3′ (forward) and 5′-GGG GTC ATT GAT GGC AAC AA-3′ (reverse). Reactions were performed using the following protocol: 50°C for 2 min, 95°C for 10 min, and 40 amplification cycles (95°C for 15 s and 60°C for 1 min), followed by a dissociation step. Melting curve analysis showed single peaks, supporting the homogeneity of amplicons. The mRNA expression levels of TNF-*α*, IL-1*β*, MCP-1, ICAM-1, and VCAM-1 relative to that of the internal control GAPDH were calculated using the comparative threshold cycle method.

### 2.7. Enzyme-Linked Immunosorbent Assays (ELISA) for Cytokines

THP-1 cells were treated with PM10 in the absence and presence of test materials for 72 h in the serum-free culture medium. The conditioned medium was collected and the concentrations of TNF-*α*, IL-1*β*, MCP-1, and ICAM-1 were measured using Human Mini ELISA kits (PeproTech, Rocky Hill, NJ, USA), according to the manufacturer's instructions. Briefly, samples (100 *μ*L conditioned medium) or solutions of standard at varied concentrations were added to microplate wells which contained immobilized capture antibody. After 12 h incubation at 4°C, the wells were washed and solutions of biotinylated detection antibody were added and incubated for 2 h. After washing the wells, solutions of horseradish peroxidase conjugated to streptavidin or avidin were added and incubated for 30 min. The cells were washed, and 3,3′,5,5′-tetramethylbenzidine (TMB) or 2,2′-Azino-bis(3-ethylbenzothiazoline-6-sulphonic acid) (ABTS) substrate solution was added to the wells to initiate enzymatic color development. The TMB reaction was terminated using 1 M HCl and the absorbance of the reaction mixture was measured at 450 nm. The absorbance of the ABTS reaction mixture was measured at 405 nm.

### 2.8. Cultivation of Endothelial Cells

The human endothelial cell line EA.hy926 purchased from American Type Culture Collection (Manassas, VA, USA) was plated on 100 mm tissue culture dishes (BD Biosciences, San Jose, CA, USA) and cultured using Dulbecco's modified Eagle's medium (DMEM) (Gibco BRL) supplemented with 10% fetal bovine serum (Gibco BRL,) and antibiotics at 37°C and 5% CO_2_.

### 2.9. Cell Adhesion Assay

Monocytic THP-1 cells were seeded in RPMI 1640 medium on 12-well tissue culture plates at 1 × 10^6^ cells/well. Cells were treated with PPE for 60 min and then with PM10 (100 *μ*g·mL^−1^) for another 24 h. Cells were collected by centrifugation and fluorescence-labeled as follows: cells were washed with phosphate buffered saline (PBS) twice, suspended at 5 × 10^6^ cells·mL^−1^ in PBS containing 5 *μ*g·mL^−1^ 2′,7′-bis(carboxyethyl)-5(6)-carboxyfluorescein acetoxymethyl ester (BCECF-AM) (Molecular Probes, Carlsbad, CA, USA), and incubated at 37°C for 60 min. The cells were collected by centrifugation, washed with PBS twice, and suspended in RPMI 1640 medium prior to being added to endothelial cell culture. EA.hy926 endothelial cells were seeded in DMEM on 6-well tissue culture plates at 2 × 10^5^ cells·cm^−2^ and cultured for 2 d. Then culture medium was replaced by RPMI 1640 medium. The fluorescence-labeled THP-1 cells were added to the EA.hy926 cell cultures at a 1 : 1 ratio. After coincubation of EA.hy926 cells and THP-1 cells in RPMI 1640 medium for 2 h, nonadherent THP-1 cells were washed twice with PBS, with caution taken not to disturb the endothelial cell monolayer. Fresh RPMI 1640 medium was supplied to the remaining cells on the culture plates. Florescence-labeled THP-1 cells adhering to the endothelial cell monolayer were observed with a Nikon eclipse TE2000-U microscope (Tokyo, Japan). For quantification, adherent cells were lysed in 200 *μ*L of 0.1 M Tris-HCl containing 0.1% Triton X-100 and centrifuged at 13,000 rpm for 15 min. The fluorescence intensity of supernatants was determined at the excitation wavelength of 485 nm and emission wavelength of 535 nm, using an LS55 fluorescence spectrometer (Perkin Elmer instruments, Waltham, MA, USA), and normalized for a number of endothelial cells.

### 2.10. Statistical Analysis 

Data are presented as the means ± standard error (SE) of three or more independent experiments. The differences between groups were statistically analyzed using Student's *t*-test, where a *p* value < 0.05 was considered statistically significant.

## 3. Results

**Figure 1:**
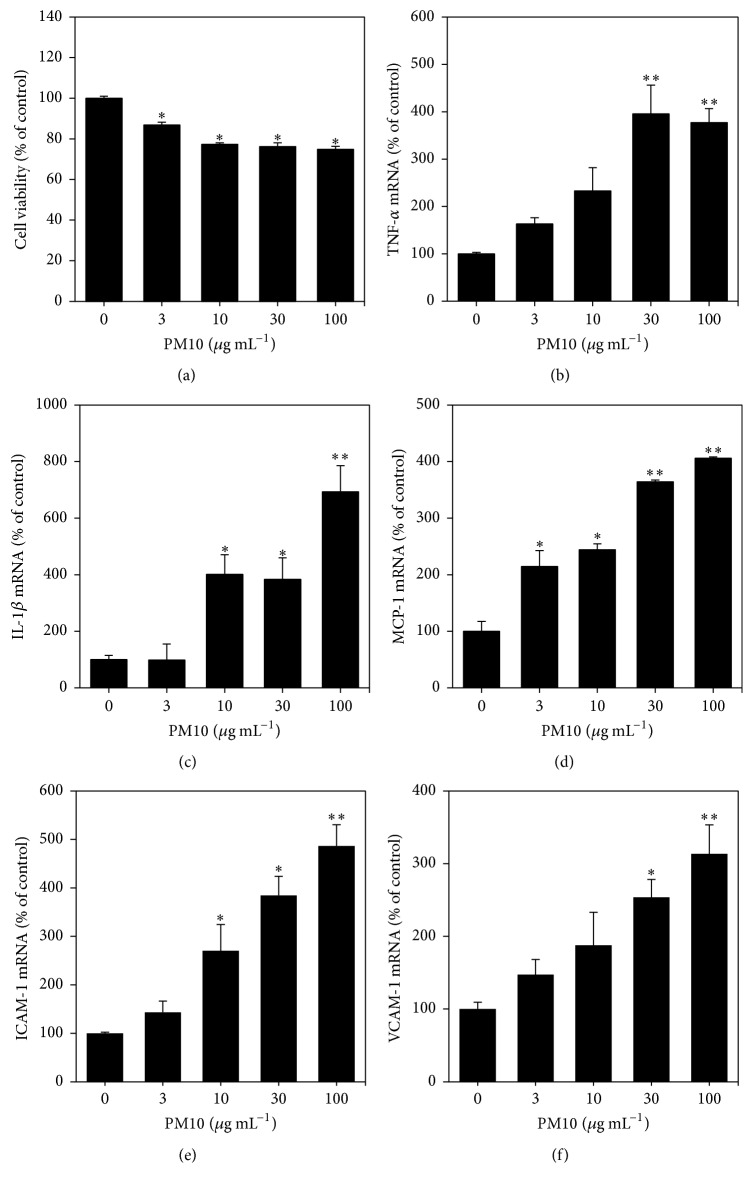
Effects of PM10 on cell viability and gene expression of inflammatory cytokines and cell adhesion molecules in THP-1 cells. Cells were treated with PM10 at the indicated concentrations for 24 h. (a) Cell viabilities are presented as percentages of viable cells per total cells. (b–f) Gene expression was analyzed by qRT-PCR and normalized to control GAPDH. Data are expressed as percentages. Data are means ± SEs (*n* = 3). ^*∗*^ *p* < 0.05 and ^*∗∗*^ *p* < 0.01 versus control.

Previous studies have shown that air-borne fine and coarse particles can cause cytotoxicity and induce proinflammatory cytokines from human monocytes [17]. In addition, it has been demonstrated that they increase the expression of cell adhesion molecules in endothelial cells [18]. Thus, we examined the cytotoxicity and proinflammatory effects of PM10 in our experimental conditions. Human monocytic THP-1 cells were treated with PM10 at various concentrations up to 100 *μ*g mL^−1^ for 24 h, and trypan blue exclusion assay was performed to determine the number of alive and dead cells. As shown in Figure 1(a), PM10 significantly decreased cell viability. To analyze gene expression, total cellular mRNA was extracted from the treated THP-1 cells, and quantitative PCR analysis was performed. The expression levels of inflammatory cytokines and cell adhesion molecules were normalized to GAPDH, a control. As shown in Figures 1(b), 1(c), and 1(d), PM10 dose-dependently increased the expression of the inflammatory cytokines TNF-*α*, IL-1*β*, and MCP-1 at the mRNA level. It also increased the expression of the cell adhesion molecules ICAM-1 and VCAM-1, as shown in Figures 1(e) and 1(f). Taken together, these data support a role of air-borne particulate matter in the induction of inflammatory reactions.

**Figure 2:**
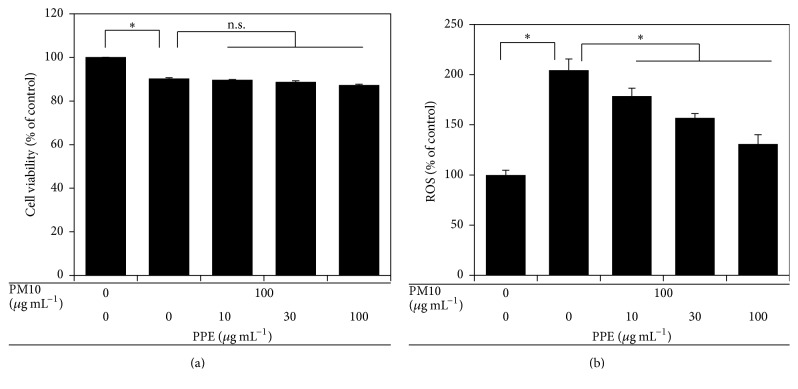
Effects of pomegranate peel extract (PPE) on cell viability and reactive oxygen species (ROS) production in THP-1 cells exposed to PM10. Cells were treated with PM10 in the absence or presence of PPE, followed by incubation for 24 h. (a) Cell viability is the percentage of viable cells out of total cells. (b) ROS production data are expressed as percentages of the control value. Data are means ± SEs (*n* = 3). ^*∗*^ *p* < 0.05; n.s., not significant.

Particulate matter induces inflammation* via* the generation of ROS and free radicals [19, 20]. Therefore, plant extracts with high contents of polyphenolic antioxidants may be protective effects against particulate matter-induced inflammation. This hypothesis was examined using PPE as a model plant extract. We determined the effects of PPE on cell viability and ROS production of THP-1 cells exposed to PM10. THP-1 cells were treated with PM10 at 100 *μ*g mL^−1^ in the absence or presence of PPE at 10–100 *μ*g mL^−1^ for 24 h. As shown in Figures 2(a) and 2(b), PM10 decreased cell viability and increased ROS production, indicating that PM10 caused oxidative stress in cells. The effect of PPE on the viability of PM10-treated cells was not significant, but it significantly and dose-dependently attenuated ROS production due to PM10 (Figures 2(a) and 2(b)). Thus, PPE can act as an antioxidant to inhibit ROS production and/or a scavenger of ROS inside cells.

**Figure 3:**
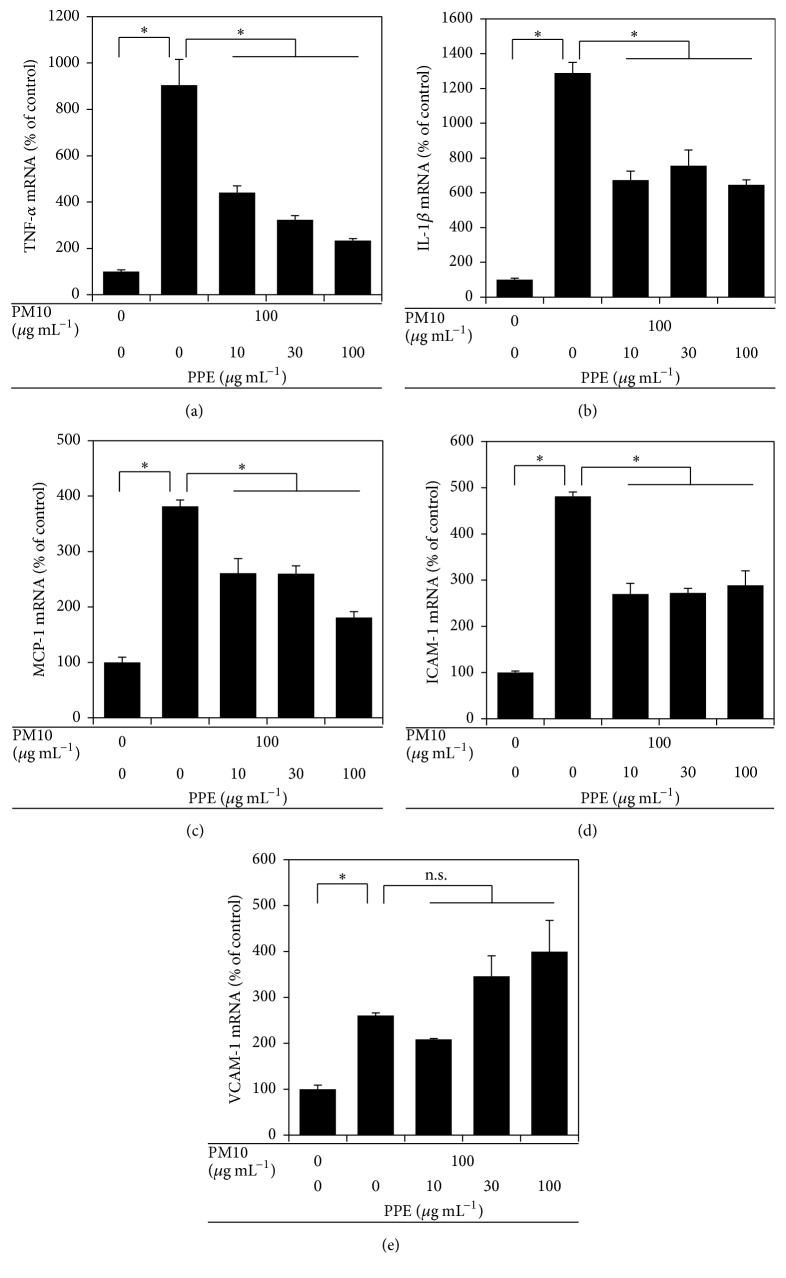
Effects of PPE on gene expression of inflammatory cytokines and cell adhesion molecules in THP-1 cells stimulated by PM10. Cells were treated with PM10 in the absence or presence of PPE, followed by incubation for 24 h. Gene expression was analyzed by qRT-PCR and normalized to control GAPDH. Data are expressed as percentages of the control value. Data are means ± SEs (*n* = 3). ^*∗*^ *p* < 0.05; n.s., not significant.

The anti-inflammatory effects of PPE were examined by monitoring the expression levels of inflammatory cytokines and cell adhesion molecules in THP-1 cells exposed to PM10. As shown in Figures 3(a)–3(c), PPE dose-dependently attenuated the expression of TNF-*α*, IL-1*β*, and MCP-1 in THP-1 cells exposed to PM10. In addition, it decreased the expression of ICAM-1 but not VCAM-1 in THP-1 cells exposed to PM10 (Figures 3(d) and 3(e)).

**Figure 4:**
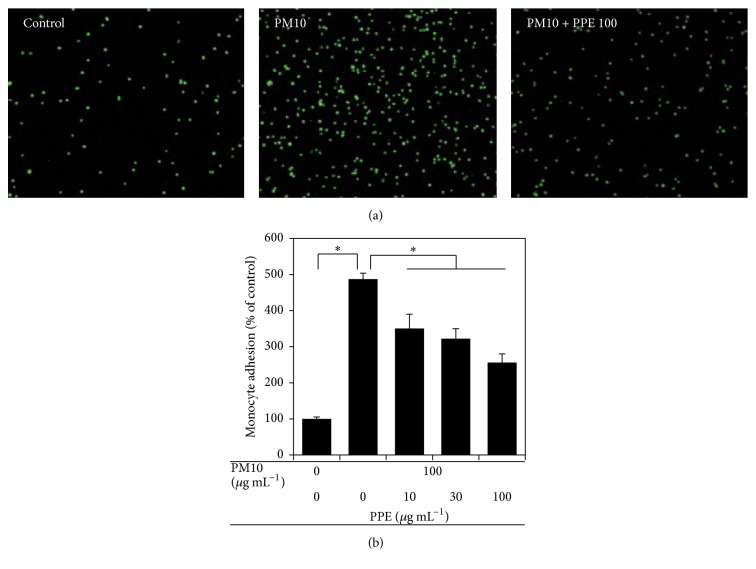
Effects of PPE on the adhesion of PM10-treated THP-1 monocytes to cells to EA.hy926 endothelial cells. THP-1 cells were treated with PM10 in the absence or presence of PPE, followed by incubation for 24 h. The treated monocytes were fluorescence-labeled and coincubated with EA.hy926 endothelial cells to monitor cell-cell adhesion. Fluorescing monocytes adhered on the endothelial cells were observed under a microscope (a) and quantified fluorometrically (b). Data are expressed as percentages of the control value. Data are means ± SEs (*n* = 3). ^*∗*^ *p* < 0.05.

The adhesion of activated monocytes to endothelial cells is a critical step of the inflammatory process, and particulate matter has been shown to increase cell adhesion [18, 21]. Thus, we examined whether PM10 activates THP-1 cells, rendering them more adhesive to endothelial cells, and whether the cell-cell interaction is attenuated by PPE. THP-1 monocytic cells were treated with PPE in the absence or presence of PPE before coincubation with EA.hy926 endothelial cells. The results showed that PM10 treatment increased adhesion of monocytes to endothelial cells, and this phenomenon was attenuated by PPE in a dose-dependent manner (Figures 4(a) and 4(b)).

**Figure 5:**
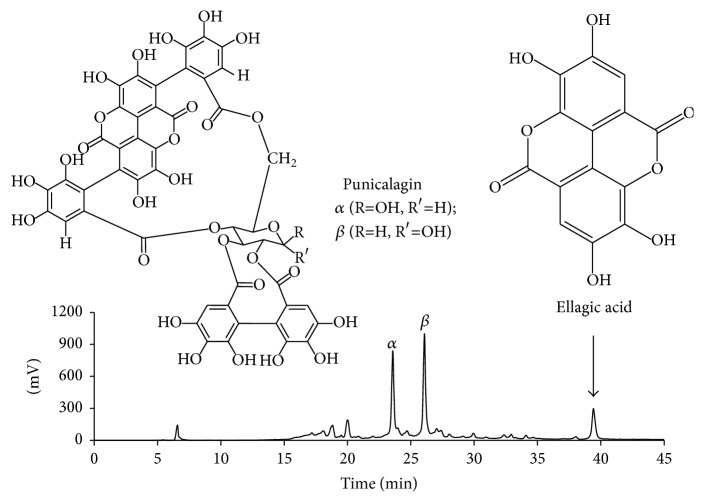
A typical high performance liquid chromatography (HPLC) chromatogram of PPE. The peaks for punicalagin and ellagic acid are indicated based on the retention times of authentic standards. Punicalagin appeared as two peaks, each corresponding to *α* and *β* anomers. Chemical structures of punicalagin *α* and *β* anomers and ellagic acid are shown.

**Figure 6:**
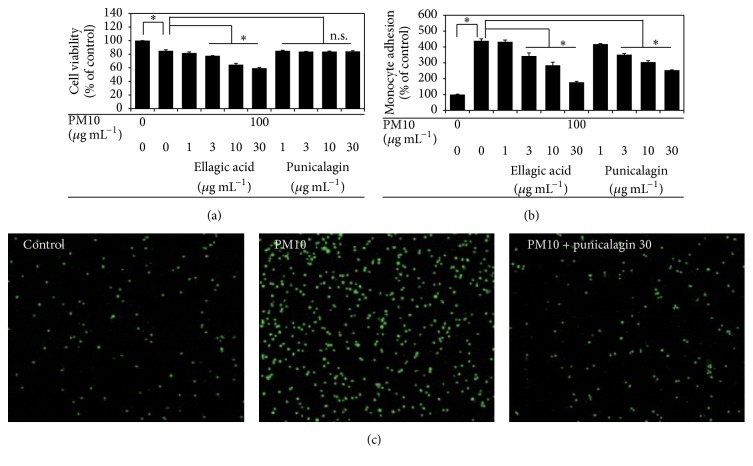
Effects of punicalagin and ellagic acid on cell viability and cell adhesiveness of THP-1 cells stimulated by PM10. THP-1 cells were treated with PM10 in the absence or presence of a test compound, followed by incubation for 24 h. Cell viabilities are presented as percentages of viable cells per total cells (a). The treated monocytes were fluorescence-labeled and coincubated with EA.hy926 endothelial cells to monitor cell-cell adhesion (b). Typical microscopic images of fluorescing monocytes adhered on the endothelial cells are shown (c). Data are expressed as percentages of the control value. Data are means ± SEs (*n* = 3). ^*∗*^ *p* < 0.05; n.s., not significant.

Ellagitannins are the major polyphenolic compounds contained in pomegranate [12]. As shown in Figure 5, HPLC analysis of PPE indicated that punicalagin and ellagic acid are major constituents. Punicalagin appeared as two peaks, each corresponding to *α* and *β* anomers. Thus, we examined if punicalagin or ellagic acid is the active constituent of PPE responsible for the anti-inflammatory effect. In this experiment, commercial forms of punicalagin and ellagic acid were tested at 1–30 *μ*g mL^−1^. As shown in Figure 6(a), ellagic acid appeared to have significant cytotoxicity, whereas punicalagin showed no cytotoxicity at the tested concentrations. Punicalagin attenuated PM10-stimulated monocyte adhesion to endothelial cells at 3–30 *μ*g mL^−1^ (Figures 6(b) and 6(c)). Ellagic acid attenuated PM10-stimulated monocyte adhesion to endothelial cells only at cytotoxic concentrations (Figure 6(b)). These data indicate that punicalagin has a better therapeutic window between efficacy concentration and toxicity concentration than ellagic acid.

**Figure 7:**
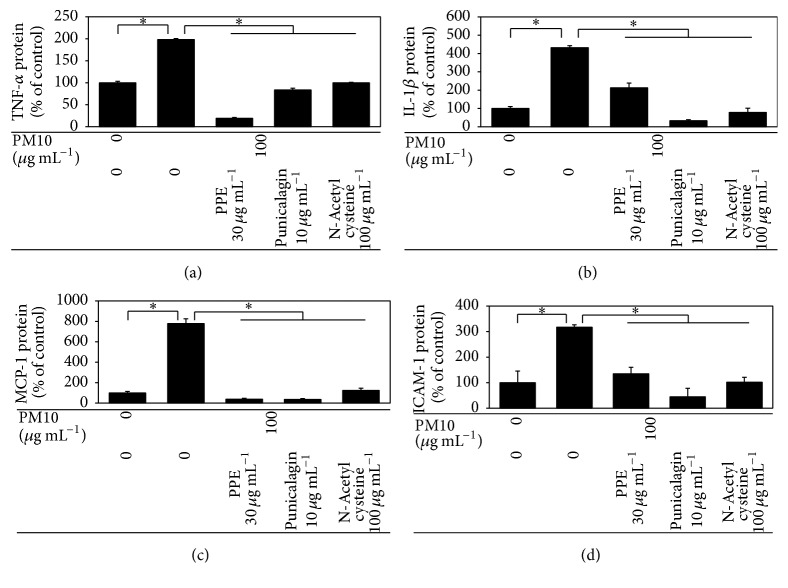
Effects of PPE, punicalagin, and N-acetyl cysteine on the levels of TNF-*α*, IL-1*β*, MCP-1, and ICAM-1 proteins released from THP-1 cells stimulated by PM10. THP-1 cells were treated with PM10 in the absence or presence of a test material for 72 h. The concentrations of TNF-*α* (a), IL-1*β* (b), MCP-1 (c), and ICAM-1 (d) proteins in the conditioned medium were measured by ELISA. Data are expressed as percentages of the control value. Data are means ± SEs (*n* = 3). ^*∗*^ *p* < 0.05.

Effects of PPE and punicalagin on the levels of TNF-*α*, IL-1*β*, MCP-1, and ICAM-1 proteins released from THP-1 cells exposed to PM10 were further examined. N-Acetyl cysteine was used as a reference antioxidant. As shown in Figure 7, PM10 elevated the secreted protein levels of TNF-*α*, IL-1*β*, MCP-1, and ICAM-1, and these changes were significantly attenuated by PPE and punicalagin as well as N-acetyl cysteine.

## 4. Discussion

The aims of this study were threefold. First aim was to examine whether PM10 stimulates inflammatory events at the cellular levels. Second aim was to examine whether such inflammatory events were attenuated by PPE. Third aim was to take insight into the active compounds of PPE.

PM10 exposure is associated with the incidence and development of cardiopulmonary disease [4, 5]. Although the precise molecular mechanisms are yet unclear, PM10 is known to stimulate alveolar macrophages and airway epithelial cells to produce inflammatory mediators such as TNF-*α* and IL-1*β* [22–24]. Particulate matter is also shown to activate endothelial cells involved in inflammation. PM10 has been shown to induce the expression of adhesion molecules and the adhesion of monocytes to human umbilical endothelial cells [18]. The mechanism of action of PM10 may include the production of ROS and activation of NF-*κ*B pathway, leading to inflammation [7]. Thus, the inflammation due to PM10 is similar to sepsis in the clinical setting [25].

As expected, the data from the current study showed that PM10 induced cytotoxicity and increased the generation of ROS, the expression of inflammatory cytokines such as TNF-*α*, IL-1*β*, and MCP-1, and the expression of cell adhesion molecules such as ICAM-1 and VCAM-1 by monocytic THP-1 cells. In addition, PM10-exposed monocytic THP-1 cells showed stronger adherences to EA.hy926 endothelial cells, supporting proinflammatory properties of PM10.

PM10-induced inflammation may be reduced by minimizing outdoor activity while atmospheric levels of particulate matter are elevated high. Additionally, certain plant extracts enriched with antioxidants are expected to reduce oxidative stress and inflammatory injury due to particulate matter. In a previous study, exposure of mice to urban air pollution increased myocardial inflammatory genes such as TNF-*α*, IL-6, and cyclooxygenase-2 (COX-2) and chocolate administration resulted in a significant downregulation of TNF-*α*, IL-6, and IL-1*β*, implicating that regular consumption of dark chocolate may reduce cardiac inflammation in the setting of air pollution exposures [9]. Another study showed that the ethanolic extract of* Eucheuma cottonii* reduced the deposition of alveolar macrophages and serum levels of malondialdehyde (MDA) in PM10 coal dust-exposed rats, indicating that the extract attenuated inflammation and oxidative stress due to chronic exposure of coal dust [10].

PPE is a well-known source of polyphenolic antioxidants and its anti-inflammatory properties have been demonstrated in various experimental models [14–16]. However, its effects on PM10-induced inflammatory responses have not been reported until the current study. The results from this study showed that PPE attenuated the PM10-induced ROS generation, expression, and secretion of TNF-*α*, IL-1*β*, MCP-1, and ICAM-1. In addition, PPE was shown to attenuate the adhesion of PM10-stimulated THP-1 cells to endothelial cells. Thus, PPE is suggested to provide health benefits by mitigating inflammatory events stimulated by particulate matter.

Literature search and HPLC analysis of PPE indicated that punicalagin and ellagic acid are major polyphenolic compounds. Thus we compared the effects of these two compounds on cell viability and cell adherence of THP-1 cells. The results indicate that punicalagin would be more useful than ellagic acid as an anti-inflammatory agent against PM10, in terms of the efficacy to safety ratios. The present study is the first to demonstrate that punicalagin (a mixture of *α* and *β* anomers) attenuates the inflammatory cytokine secretion and cell adhesion of monocytic cells stimulated with airborne dust, although previous studies have reported that punicalagin can provide health benefits in various other experimental conditions [26–28]. Previous studies have shown that punicalagin is highly bioavailable and safe in animal models [29, 30]. Punicalagin is metabolized to punicalin, gallagic acid, and ellagic acid [31]. Therefore, not only does punicalagin offer antioxidant and anti-inflammatory effects on its own, but its metabolite can provide similar effects in the body.

Although the present study clearly demonstrated that PM10-induced inflammation can be attenuated by PPE at the cellular levels and identified punicalagin as an active constituent of PPE, the health benefits of PPE and punicalagin remain to be validated in additional* in vivo* studies.

In conclusion, we demonstrated that PPE prevented inflammatory events due to particulate matter. PPE attenuated ROS production, the expression of inflammatory cytokines, and cell adhesion molecules in THP-1 monocytic cells exposed to PM10. PPE also decreased the cell-cell adhesion between PM10-stimulated THP-1 cells and EA.hy926 endothelial cells. Punicalagin appeared to attenuate the cell-cell adhesion between PM10-stimulated THP-1 cells and EA.hy926 endothelial cells, without cytotoxicity. These results support the protective effects of PPE and punicalagin against oxidative stress and inflammatory responses induced by harmful airborne dust.
